# Kidney Transplantation From Donors With Acute Kidney Injury: Are the Concerns Justified? A Systematic Review and Meta-Analysis

**DOI:** 10.3389/ti.2023.11232

**Published:** 2023-05-18

**Authors:** George Emilian Nita, Jeevan Prakash Gopal, Hussein A. Khambalia, Zia Moinuddin, David van Dellen

**Affiliations:** ^1^ Department of Renal and Pancreas Transplantation, Manchester Royal Infirmary, Manchester University NHS Foundation Trust, Manchester, United Kingdom; ^2^ Faculty of Biology, Medicine and Health, University of Manchester, Manchester, United Kingdom

**Keywords:** delayed graft function, acute kidney injury, primary non-function, donors and donation, graft outcome

## Abstract

Renal transplantation improves quality of life and prolongs survival in patients with end-stage kidney disease, although challenges exist due to the paucity of suitable donor organs. This has been addressed by expanding the donor pool to include AKI kidneys. We aimed to establish whether transplanting such kidneys had a detrimental effect on graft outcome. The primary aim was to define early outcomes: delayed graft function (DGF) and primary non-function (PNF). The secondary aims were to define the relationship to acute rejection, allograft survival, eGFR and length of hospital stay (LOS). A systematic literature review and meta-analysis was conducted on the studies reporting the above outcomes from PubMed, Embase, and Cochrane Library databases. This analysis included 30 studies. There is a higher risk of DGF in the AKI group (OR = 2.20, *p* < 0.00001). There is no difference in the risk for PNF (OR 0.99, *p* = 0.98), acute rejection (OR 1.29, *p* = 0.08), eGFR decline (*p* = 0.05) and prolonged LOS (*p* = 0.11). The odds of allograft survival are similar (OR 0.95, *p* = 0.54). Transplanting kidneys from donors with AKI can lead to satisfactory outcomes. This is an underutilised resource which can address organ demand.

## Graphical Abstract

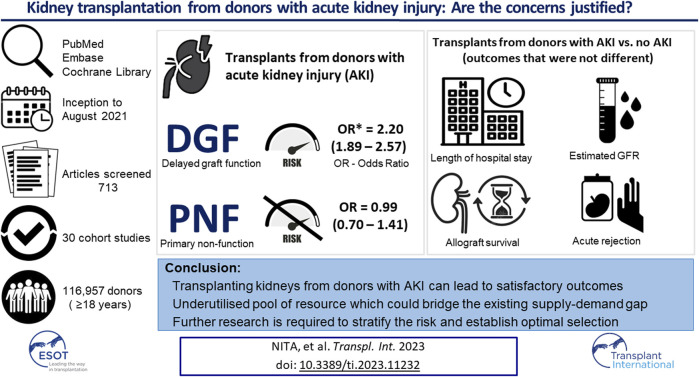

## Introduction

Globally, the prevalence of chronic kidney disease (CKD) is approximately 13% [1]. Renal transplantation is a well-established safe procedure, shown to improve the quality of life (QoL) and prolong the life expectancy of CKD patients requiring renal replacement therapy (RRT) compared to dialysis [2–4]. There is an increasing demand for organs available for transplantation. The field of organ transplantation is constantly evolving and strategies such as expansion of the donor pool to include organs from donors after circulatory death (DCD) and the introduction of extended criteria donors (ECD) were adopted globally to address the disparity between organ supply and demand [5–7]. An additional strategy to overcome organ shortage is the utilisation of AKI donor kidneys. Despite this, the supply-demand mismatch remains significant. In 2019–2020 over 4,000 patients were active on the UK renal transplantation waiting list and less than 2,500 kidney transplants from deceased donors were performed nationally [8]. The waiting list mortality remains significant with 1-year and 3-year mortality reaching 2% and 4% in the UK. In the USA, the mortality rate has increased to 5.7 deaths per 100 waitlist years, the highest since 2012 [9, 10].

The growing gap between supply and demand is exacerbated by discarding potentially usable organs. In the US, approximately 20% of the deceased donor kidneys are discarded annually [11, 12]. The discard rate for AKI donors remains high, reaching approximately 10% in the UK and 25% in the US [13]. Despite the introduction of the Kidney Donor Profile Index (KDPI) as a new allocation system in the US, the discard rate remains high [14].

This study aims to establish whether transplanting AKI donor kidneys has a detrimental effect on graft outcome, and subsequently to determine if AKI kidneys are a reasonable and safe option to address the organ shortage.

## Materials and Methods

A meta-analysis was conducted according to the Preferred Reporting Items for Systematic Reviews and Meta-analysis (PRISMA) guidelines [15]. Three databases were selected for literature search: PubMed (Medline), Embase (Ovid) and the Cochrane Library. To assess the quality of the studies included in this meta-analysis, the Newcastle-Ottawa Scale (NOS) and the Oxford Centre for Evidence-Based Medicine (OCEBM) hierarchy were utilised [16, 17].

### Search Strategy

The search strategy included the terms “renal” or “kidney transplantation,” “donor” or “donors” and “acute kidney injury” or “AKI.” Databases were searched from inception to 1 May 2022. Two independent reviewers (GN and JG) performed a full-text screening of the studies. A third reviewer (DVD) resolved any conflicts.

- Medline (PubMed): (“renal transplantation”[tiab] OR “kidney transplantation[tiab]”) AND (“acute kidney injury”[tiab] OR ‘AKI'[tiab]) AND (“donor”[tiab] OR “donors”[tiab])
- Embase (Ovid): ((renal transplantation or kidney transplantation) and (acute kidney injury or AKI) and (donor or donors)) ab,ti.
- Cochrane Library: (renal transplantation OR kidney transplantation) AND (acute kidney injury OR AKI) AND (donor OR donors) in Title Abstract Keyword - (Word variations have been searched)

### Inclusion and Exclusion Criteria

The aim was to identify all prospective studies (cohort studies or randomised control trials) performed in the adult population (≥18 years), reporting renal graft function, acute rejection, and graft survival, and comparing the outcome between donors with AKI versus non-AKI donors, from inception to May 2022 across PubMed (Medline), Embase (Ovid), and the Cochrane Library databases.

The inclusion criteria were defined as:

• studies reporting on adult patients (≥18 years of age)
• studies referring to patients receiving a renal transplantation as the primary and single transplant procedure AND
• comparing and reporting outcomes in the AKI and non-AKI donor groups
• articles fully accessible AND
• written in English

The exclusion criteria were defined as:

• studies reporting outcomes in the paediatric population (<18 years)
• studies comparing donors after brain death (DBD) with donors after circulatory death (DCD)
• studies reporting on simultaneous kidney pancreas (SPK) transplants or kidney re-transplantation/secondary transplant procedure
• studies on animal models
• studies lacking a control group
• case-series or low number studies (<50)
• abstracts-only available; letters or reviews
• full text not accessible or not available in English

### Data Extraction and Quality Assessment

Data extracted from each study included: the first author name and publication year, country of origin, the study period and study design, the number of donors included, criteria utilised to define and classify AKI, mean donor age, gender, follow up period, and the reported endpoints (delayed graft function (DGF), primary non-function (PNF)), acute rejection, graft survival, eGFR at 1 year and duration of hospital stay). The Oxford Centre for Evidence-Based Medicine (OCEBM) 2011 Levels of Evidence hierarchy [16] and the 9-point Newcastle-Ottawa scale (NOS) were utilised to assess the level of evidence and quality of the studies included in the meta-analysis [17].

**FIGURE 1:**
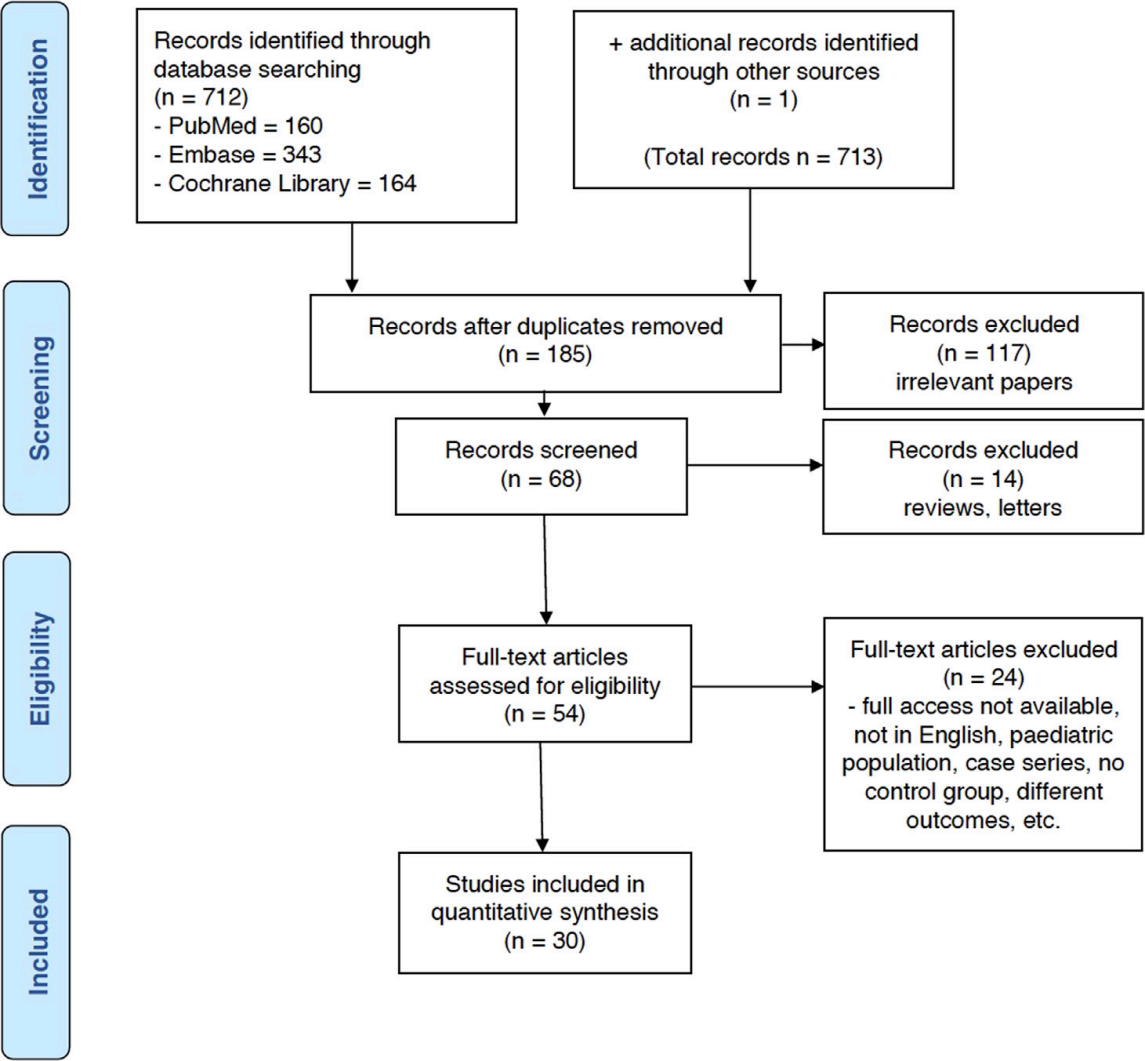
PRISMA flowchart.

The initial search across the three databases returned 712 records (PubMed—160; Embase—343; Cochrane Library—164). 1 additional record was manually added (total *n* = 713). After the initial screening, 185 duplicate records were removed and 117 records were excluded. 68 records were further screened and 14 were further excluded (reviews and letters). 54 full-text articles were assessed for eligibility. Articles which could not be fully accessed, not written in English, reporting outcomes in the paediatric population, case series, reporting on different outcomes or lacking a control group (i.e., 24 records) were excluded. Finally, 30 studies were included comprising of 116,957 donors. This is illustrated in the PRISMA flowchart (Figure 1).

Five studies reported outcomes in two different groups: extended versus standard criteria donors—Kayler et.al. [18], Jacobi et al. [19], Heilman et al. [20], Ko et al. [21], and low versus high KDPI (Kidney Donor Profile Index)—Park et al. [22]. For these studies data was analysed separately comparing the outcomes for each subgroup of patients. The acceptable follow up period was established as 12 months post-transplantation for the study endpoints. Adequacy of follow-up was scored only where the follow up was complete and all the subjects were accounted for. No points were allocated for adequacy if the follow up rate was <80%, there was no description for lost to follow up patients or no statement with regards to follow up was made by the authors.

The primary outcome of this systematic review and meta-analysis was to determine the effect of transplanting AKI kidneys on the early graft function: delayed graft function (DGF) and primary non-function (PNF). The secondary aims were to determine the relationship between transplanting AKI donor kidneys and: acute rejection (AR), allograft survival, eGFR at 1-year post-transplantation, and length of hospital stay (LOS).

### Data Analysis and Statistical Tests

The data was collated and analysed using Review Manager (RevMan) [Computer program]. Version 5.4. The Cochrane Collaboration, 2020.

Odds ratios (ORs) of every outcome and the 95% confidence intervals (CIs) were calculated for the dichotomous data (DGF, PNF, acute rejection and allograft survival). For the continuous data (eGFR and length of hospital stay), the weighted mean difference (WMD) and 95% confidence intervals (CI) were calculated. An estimate of the between-study variance was reported using the tau-squared (τ^2^/Tau^2^) and the Chi-squared (Chi^2^) tests to assess whether the differences were due to chance. Accompanying *p* values were calculated for the heterogeneity tests. To quantify the percentage of variation due to heterogeneity the *I* ^*2*^ test was used. Thresholds for the interpretation of *I* ^*2*^ were established as per the Cochrane Handbook for Systematic Reviews of Interventions, Version 6.2, 2021 (“0%–40%: might not be important; 30%–60%: may represent moderate heterogeneity; 50%–90%: may represent substantial heterogeneity; 75%–100%: considerable heterogeneity”) [23]. The random-effects model (Mantel-Haenszel method and the inverse variance methods) was chosen for this meta-analysis. The Z test was used for the pooled overall effect.

## Results

### Study Characteristics & Quality Assessment

All the studies included in this meta-analysis were cohort studies (single centre, multi-centre, and National Transplant Registry analyses) from Europe, North America, Australia, and Asia. The study periods ranged between 1995 and 2017 with a follow-up period ranging between 12 months and 132 months. The main study characteristics are illustrated in Table 1.

**TABLE 1:** Main study characteristics.

	Author	Year	Country	Study design	Study period	AKI criteria	Total no. of donors (n)	Mean donor age	Donor gender (M:F ratio)	Follow up (months)	Endpoints
1	Kayler et al.* (SCD) [18]	2009	USA	Cohort study^a^	1995–2007	tSCr	48,558	37	-	120	DGF allograft survival
	Kayler et al.* (ECD) [18]	2009	USA	Cohort study^a^	1995–2007	tSCr	17,051	37	-	120	DGF allograft survival
2	Rodrigo et al. [24]	2010	Spain	Cohort study^b^	1994–2006	RIFLE	176	AKI: 46.3 ± 13.2 non-AKI: 45.8 ± 16.7	AKI: 1.7:1 non-AKI: 1.2:1	-	DGF acute rejection allograft survival
3	Kolonko et al. [25]	2011	Poland	Cohort study^b^	1996–2006	RIFLE	61	AKI: 50 non-AKI: 43	AKI: 1.5:1 non-AKI: 2.4:1	49 ± 18	DGF allograft survival
4	Farney et al. [26]	2013	USA	Cohort study^b^	2007–2012	tSCr	367	AKI: 36 ± 13 non-AKI: 35 ± 15	AKI: 3.2:1 non-AKI: 1.2:1	35 (6–70)	DGF PNF allograft survival
5	Jung et al. [27]	2013	Korea	Cohort study^b^	2009–2012	RIFLE	54	AKI: 45.67 ± 14.27 non-AKI: 50.39 ± 25.18	AKI: 8:1 non-AKI: 1.6:1	23.2 ± 10.4	DGF
6	Jacobi et al.* (SCD) [19]	2014	Germany	Cohort study^b^	2008–2014	RIFLE	208	AKI: 42.5 ± 12.6 non-AKI: 39.5 ± 11.8	AKI: 2.7:1 non-AKI: 0.9:1	12	DGF PNF allograft survival + eGFR + hospital stay
	Jacobi et al.* (ECD) [19]	2014	Germany	Cohort study^b^	2008–2014	RIFLE	174	AKI: 66.9 ± 9.5 non-AKI: 67.7 ± 6.9	AKI: 1.6:1 non-AKI: 0.8:1	12	DGF PNF allograft survival + eGFR + hospital stay
7	Lee et al. [28]	2014	Korea	Cohort study^b^	1996–2012	AKIN	156	AKI: 43.3 ± 13.8 non-AKI: 41.1 ± 14.6	AKI: 0.3:1 non-AKI: 2.3:1	12	DGF allograft survival + eGFR
8	Yu et al. [29]	2014	China	Cohort study^b^	2005–2011	RIFLE	57	AKI: 40 ± 9.8 non-AKI: 35 ± 12.2	AKI: 2.8:1 non-AKI: 2.5:1	12	DGF acute rejection + eGFR
9	Yuan et al. [30]	2014	China	Cohort study^b^	2011–2013	RIFLE	89	AKI: 37 ± 15.2 non-AKI: 37.5 ± 13.5	AKI: 2.3:1 non-AKI: 4:1	18 (7–26)	DGF acute rejection + eGFR
10	Molina et al. [31]	2015	Spain	Cohort study^b^	1976–2013	tSCr	118	AKI: 52 ± 13 non-AKI: 50 ± 13	AKI: 1.1:1 non-AKI: 1.1:1	AKI: 101 mo ± 67	DGF allograft survival
										Non-AKI: 99 mo ± 70	
11	Ali et al. [32]	2015	Saudi Arabia	Cohort study^b^	2000–2012	AKIN	261	AKI: 36.7 ± 11.0 non-AKI: 35.0 ± 13.0	AKI: 4.6:1 non-AKI: 10:1	120	DGF acute rejection allograft survival + eGFR
12	Benck et al. [33]	2015	Germany	Cohort study^b^	-	RIFLE	98	AKI: 53 ± 13 non-AKI: 54.8 ± 15.5	AKI: 3.1:1 (25/8)	-	DGF allograft survival
									Non-AKI: 0.8:1 (28/37)		
13	Hall et al. [34]	2015	USA	Cohort study^c^	2010–2013	AKIN	1,369	AKI: 39 non-AKI: 41	AKI: 1.7:1 non-AKI: 1.5:1	20 (11.5–28.5)	DGF
14	Heilman et al.* (SCD) [20]	2015	USA	Cohort study^b^	2004–2013	AKIN	621	AKI: 32.3 ± 13.2 non-AKI: 34.5 ± 15.4	AKI: 3.5:1 non-AKI: 1.6:1	19.6–41.4	DGF acute rejection allograft survival + eGFR + hospital stay
	Heilman et al.* (ECD) [20]	2015	USA	Cohort study^b^	2004–2013	AKIN	160	AKI: 56.6 ± 9.1 non-AKI: 61.6 ± 9.2	AKI: 2.8:1 non-AKI: 1:1	12.3–23.8	DGF acute rejection allograft survival eGFR hospital stay
15	Wiwattanathum et al. [35]	2016	Thailand	Cohort study^b^	2012–2013	AKIN	111	AKI: 43.9 ± 12.0 non-AKI: 42.9 ± 19.9	AKI: 2.2:1 non-AKI: 1.1:1	48	DGF
16	Boffa et al. [13]	2017	UK	Cohort study^a^	2003–2013	AKIN	11,219	-	AKI: 1.8:1 non-AKI: 1:1	12	DGF PNF allograft survival
17	Kim et al. [36]	2017	Korea	Cohort study^b^	1996–2014	KDIGO AKIN	285	AKI: 49.1 ± 11.3 non-AKI: 46.5 ± 8.0	AKI: 1:1 non-AKI: 1.3:1	-	DGF allograft survival eGFR
18	Bauer et al. [37]	2018	Germany	Cohort study^b^	2005–2016	pSCr	642	AKI: 49.31 ± 16.34 non-AKI: 55.28 ± 16.08	AKI: 3.7:1 non-AKI: 0.6:1	55.82 ± 34.97	DGF PNF acute rejection allograft survival eGFR
19	Yeon et al. [38]	2018	Korea	Cohort study^b^	2005–2014	KDIGO	413	AKI: 45 [35–56] non-AKI: 48 [35–55]	AKI: 1.7:1 non-AKI: 1.9:1	52.8	DGF acute rejection allograft survival
20	Ko et al.* (SCD) [21]	2018	Korea	Cohort study^b^	2000–2013	AKIN	149	AKI: 38.5 ± 10.0 non-AKI: 39.4 ± 14.9	AKI: 3.5:1 non-AKI: 1.6:1	40.3	DGF allograft survival eGFR
	Ko et al.* (ECD) [21]	2018	Korea	Cohort study^b^	2000–2013	AKIN	53	AKI: 56.7 ± 6.1 non-AKI: 58.4 ± 4.7	AKI: 3.5:1 non-AKI: 1.2:1	40.3	DGF allograft survival eGFR
21	Gwon et al. [39]	2018	Korea	Cohort study^b^	2009–2015	AKIN	101	AKI: 46.2 ± 13.5 non-AKI: 51.0 ± 20.1	AKI: 7:1 non-AKI: 1.3:1	-	DGF allograft survival eGFR
22	Torabi et al. [40]	2019	USA	Cohort study^b^	2014–2016	AKIN	285	AKI: 56.1 ± 13.7 non-AKI: 56.9 ± 12.1	AKI: 1.8:1 non-AKI: 2:1	-	DGF acute rejection allograft survival eGFR
23	Domagala et al. [41]	2019	Poland	Cohort study^b^	2010–2011	tSCr	226	AKI: 42 ± 15 non-AKI: 47 ± 15	AKI: 4:1 non-AKI: 1.2:1	60	DGF acute rejection allograft survival hospital stay
24	Hall et al. [42]	2019	USA	Cohort study^c^	2010–2013	AKIN	1,298	AKI: 41 ± 14 non-AKI: 42 ± 15	AKI: 1.7:1 non-AKI: 1.5:1	48	allograft survival
25	Schütte-Nütgen et al. [43]	2019	Germany	Cohort study^b^	2004–2014	AKIN	214	AKI: 54.3 ± 17.2 non-AKI: 51.1 ± 16.5	AKI: 1.4:1 non-AKI: 1.3:1	60	DGF allograft survival eGFR
26	van der Windt et al. [44]	2019	USA	Cohort study^b^	2013–2017	AKIN	333	AKI: 41.5 ± 12.9 non-AKI: 41.3 ± 13.7	AKI: 1.3:1 non-AKI: 1.7:1	32	DGF acute rejection allograft survival eGFR
27	Liu et al. [45]	2020	USA	Cohort study^a^	2010–2013	KDIGO	25,323	AKI: 42 (28–52) non-AKI: 42 (27–52)	AKI: 1.7:1 non-AKI: 1.7:1	60	DGF PNF allograft survival eGFR
28	Pei et al. [46]	2021	Australia & New Zealand	Cohort study^a^	1997–2017	KDIGO	5,744	AKI: 46 (30–58) non-AKI: 46 (30–58)	AKI: 2:1 non-AKI: 1.2:1	62 (24–114)	DGF acute rejection allograft survival
29	Kim et al. [14]	2021	Korea	Cohort study^b^	2003–2016	KDIGO	376	AKI: 47.9 ± 14.1 non-AKI: 44.2 ± 16.0	AKI: 2.1:1 non-AKI: 1.8:1	AKI: 78 (51–103) non-AKI: 96 (63–132)	DGF acute rejection allograft survival eGFR
30	Park et al.** (lKDPI) [22]	2021	Korea	Cohort study^c^	1996–2017	KDIGO	269	AKI: 36.4 ± 10.7 non-AKI: 34.8 ± 13.7	AKI: 3.6:1 non-AKI: 2.3:1	48 (22.3–68)	DGF acute rejection allograft survival eGFR
	Park et al.** (hKDPI) [22]	2021	Korea	Cohort study^c^	1996–2017	KDIGO	338	AKI: 54.5 ± 8.3 non-AKI: 56.2 ± 10.0	AKI 2.5:1 non-AKI: 1.2:1	48 (22.3–68)	DGF acute rejection allograft survival eGFR

^a^ National Transplant Registry analysis.

^b^ Single-centre cohort study.

^c^ Multi-centre cohort study.

*Standard Criteria Donors (SCD); Extended Criteria Donors (ECD).

**Low Kidney Donor Profile Index (lKDPI); High Kidney Donor Profile Index (hKDPI).

AKI, Acute Kidney Injury; tSCr, (donor) terminal Serum Creatinine; pSCr—(donor) peak Serum Creatinine; DGF, Delayed Graft Function; PNF, Primary Non-Function; eGFR, estimated Glomerular Filtration Rate.

The included studies correspond to Level 2 on the Oxford CEBM 2011 hierarchy [16]. The studies were assessed for quality according to the 9-point NOS (Table 2). Studies scoring 7 or greater on the NOS scale were regarded as good quality studies.

**TABLE 2:** Quality assessment of non-randomised cohort studies (Newcastle-Ottawa Scale).

	Author (year)	Representativeness of the exposed cohort	Selection of the non-exposed cohort	Ascertainment of exposure	Demonstration outcome of interest not present at start	Comparability of cohorts	Assessment of outcome	Follow-up period	Follow-up adequacy	Total (out of 9)
1	Kayler et al. [18]	⋆	⋆	⋆	⋆	⋆	⋆	⋆	⋆	8
2	Rodrigo et al. [24]	⋆	⋆	⋆	⋆		⋆		⋆	6
3	Kolonko et al. [25]	⋆	⋆	⋆	⋆	⋆	⋆	⋆	⋆	8
4	Farney et al. [26]	⋆	⋆	⋆	⋆		⋆	⋆	⋆	7
5	Jung et al. [27]	⋆	⋆	⋆	⋆	⋆	⋆	⋆		7
6	Jacobi et al. [19]	⋆	⋆	⋆	⋆		⋆	⋆		6
7	Lee et al. [28]	⋆	⋆	⋆	⋆	⋆	⋆	⋆		7
8	Yu et al. [29]	⋆	⋆	⋆	⋆		⋆	⋆	⋆	7
9	Yuan et al. [30]	⋆	⋆	⋆	⋆	⋆	⋆	⋆		7
10	Molina et al. [31]	⋆	⋆	⋆	⋆	⋆	⋆	⋆		7
11	Ali et al. [32]	⋆	⋆	⋆	⋆	⋆	⋆	⋆	⋆	8
12	Benck et al. [33]	⋆	⋆	⋆	⋆	⋆	⋆			6
13	Hall et al. [34]	⋆	⋆	⋆	⋆	⋆	⋆	⋆	⋆	8
14	Heilman et al. [20]	⋆	⋆	⋆	⋆	⋆	⋆	⋆		7
15	Wiwattanathum et al. [35]	⋆	⋆	⋆	⋆	⋆	⋆	⋆		7
16	Boffa et al. [13]	⋆	⋆	⋆	⋆	⋆	⋆	⋆		7
17	Kim et al. [36]	⋆	⋆	⋆	⋆	⋆⋆	⋆	⋆		8
18	Bauer et al. [37]	⋆	⋆	⋆	⋆	⋆	⋆	⋆		7
19	Yeon et al. [38]	⋆	⋆	⋆	⋆	⋆	⋆	⋆		7
20	Ko et al. [21]	⋆	⋆	⋆	⋆	⋆	⋆	⋆	⋆	8
21	Gwon et al. [39]	⋆	⋆	⋆	⋆		⋆	⋆		6
22	Torabi et al. [40]	⋆	⋆	⋆	⋆	⋆⋆	⋆	⋆		8
23	Domagala et al. [41]	⋆	⋆	⋆	⋆		⋆	⋆	⋆	7
24	Hall et al. [42]	⋆	⋆	⋆	⋆		⋆	⋆	⋆	7
25	Schütte-Nütgen et al. [43]	⋆	⋆	⋆	⋆		⋆	⋆	⋆	7
26	van der Windt et al. [44]	⋆	⋆	⋆	⋆	⋆	⋆	⋆	⋆	8
27	Liu et al. [45]	⋆	⋆	⋆	⋆	⋆⋆	⋆	⋆	⋆	9
28	Pei et al. [46]	⋆	⋆	⋆	⋆	⋆⋆	⋆	⋆	⋆	9
29	Kim et al. [14]	⋆	⋆	⋆	⋆	⋆⋆	⋆	⋆		8
30	Park et al. [22]	⋆	⋆	⋆	⋆	⋆	⋆	⋆		7

Maximum of ⋆ awarded for each item except for “comparability” where a maximum of ⋆⋆ can be awarded.

A study scoring 7 and above was regarded as a good quality cohort study.

Acceptable follow up period was established at least 12 months for the endpoints.

### Primary Outcomes

#### Delayed Graft Function (DGF)

**FIGURE 2:**
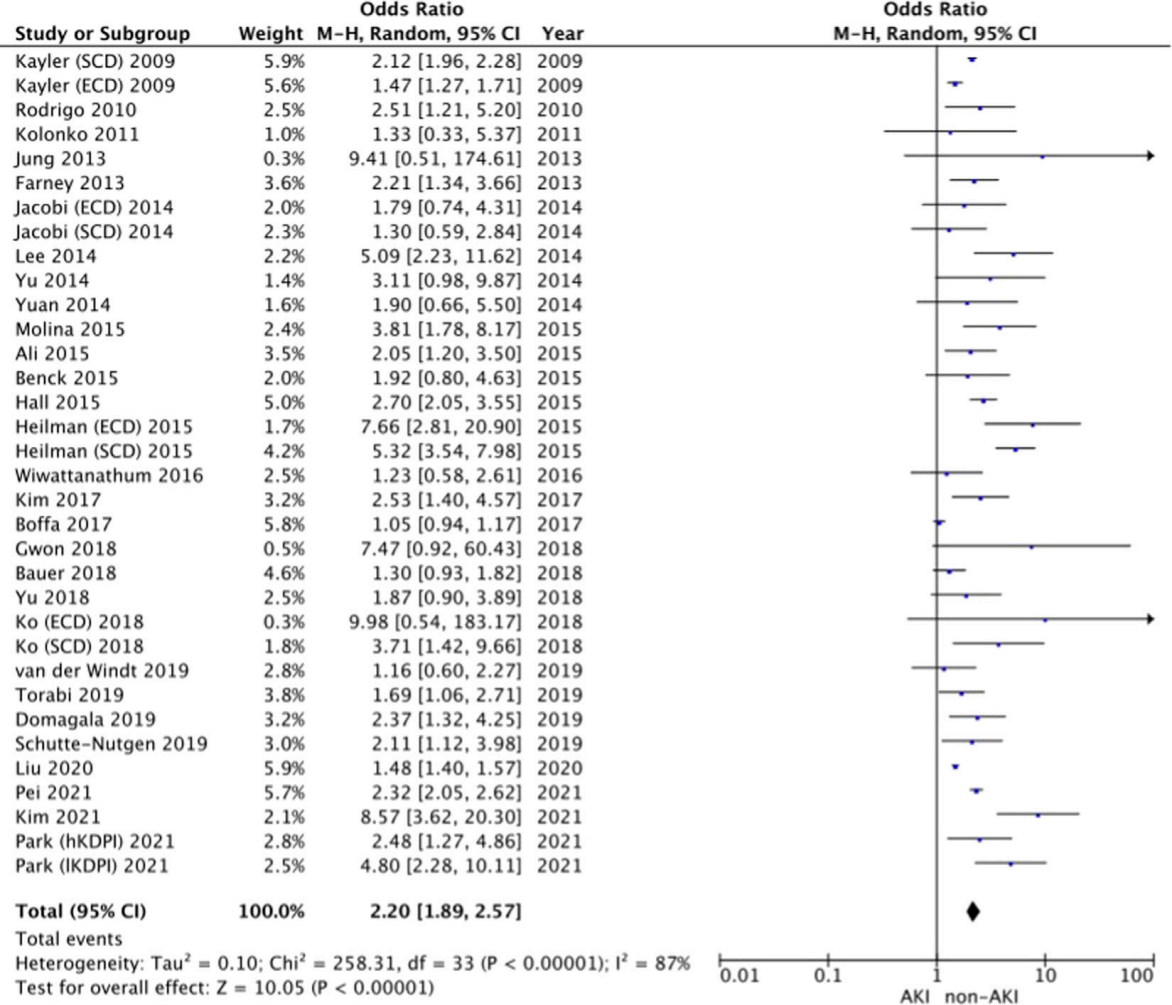
Delayed graft function—Forrest plot.

29 studies included in this meta-analysis reported on the incidence of DGF in the donor AKI versus the non-AKI groups [13, 14, 18–22, 24–41, 43–46]. The pooled odds of DGF are higher in the AKI group vs. the non-AKI group (OR = 2.20, 95% CI = 1.89–2.57, I^2^ = 87%, Z = 10.05, *p* < 0*.*00001 (Figure 2).

#### Primary Non-Function (PNF)

**FIGURE 3:**
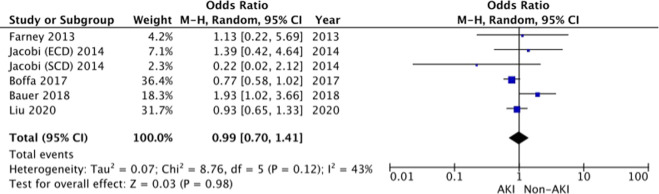
Primary non-function—Forrest plot.

5 studies: Farney et al. [26], Jacobi et al [19], Boffa et al. [13], Bauer et al [37] and Liu et al. [45], reported the incidence of PNF. The pooled result demonstrates no significant difference in the odds of developing PNF in AKI versus the non-AKI groups (OR 0.99, 95% CI = 0.70–1.41, I^2^ = 43%, Z = 0.03, *p* = 0.98) (Figure 3).

### Secondary Outcomes

#### Acute Rejection

**FIGURE 4:**
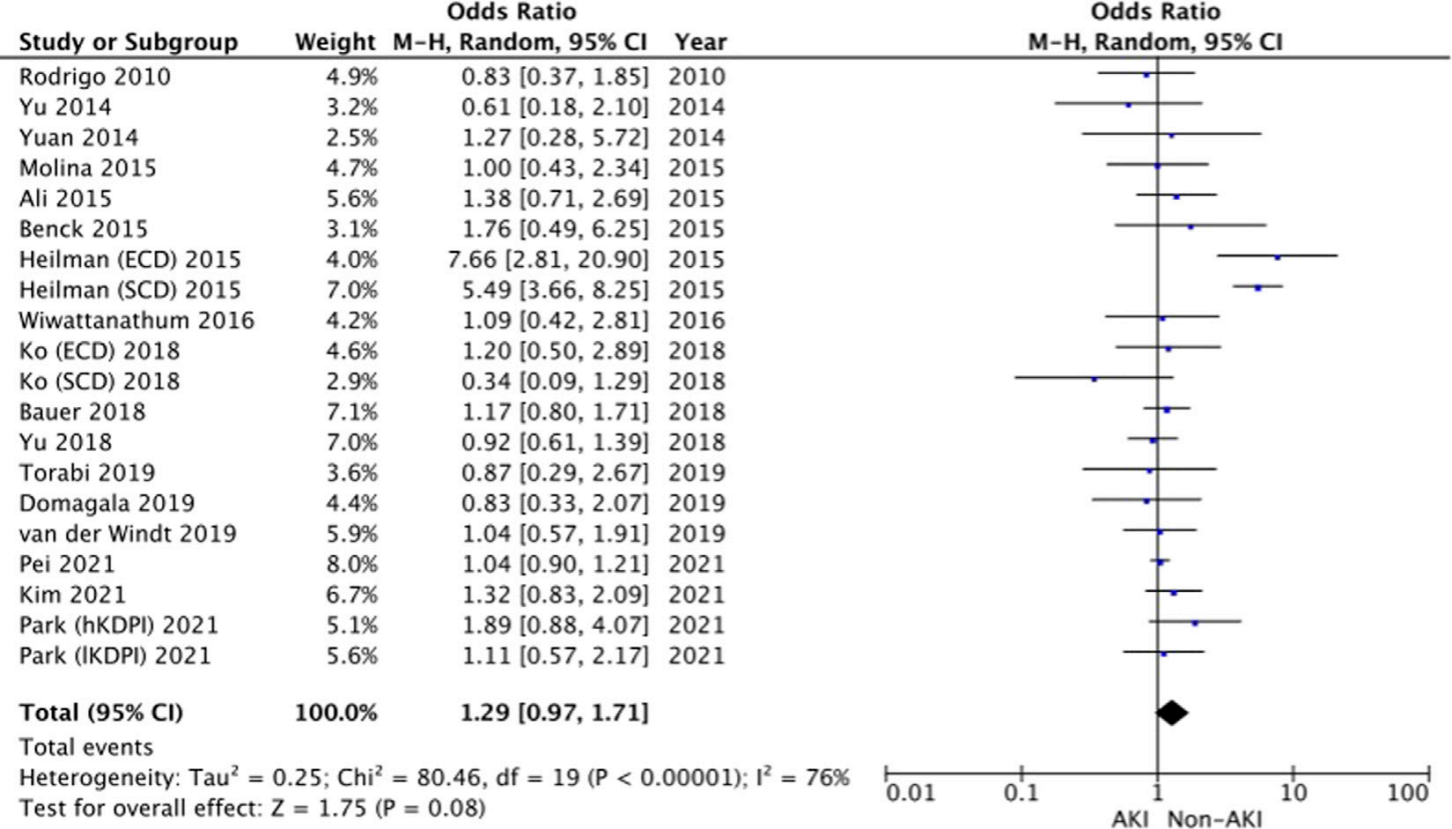
Acute rejection—Forrest plot.

Data from 17 studies [14, 20–22, 24, 29–33, 35, 37, 38, 40, 41, 44, 46] reporting acute rejection was pooled. The results show no significant difference in the odds of acute rejection between donor AKI vs. non-AKI kidneys groups (OR 1.29, 95% CI = 0.97–1.71, I^2^ = 76%, Z = 1.75, *p* = 0.08). (Figure 4).

#### Allograft Survival

**FIGURE 5:**
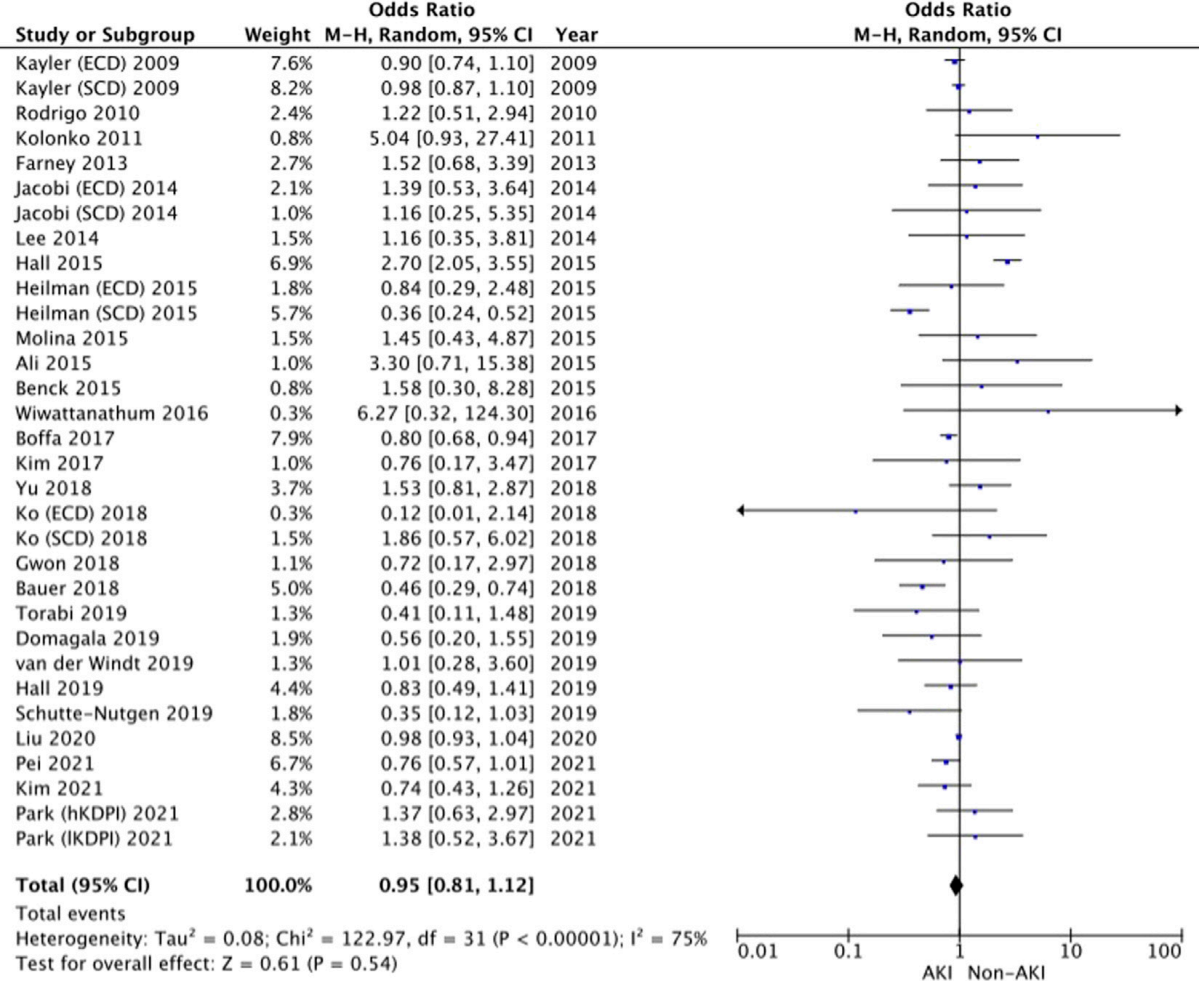
Allograft survival–Forrest plot.

27 studies reported on allograft survival [13, 14, 18–22, 24–26, 28, 31–46]. The Forrest plot demonstrates similar odds of allograft survival between the two groups (OR 0.95, 95% CI = 0.81–1.12, I^2^ = 75%, Z = 0.61, *p* = 0.54). (Figure 5).

#### Estimated Glomerular Filtration Rate (eGFR)

**FIGURE 6:**
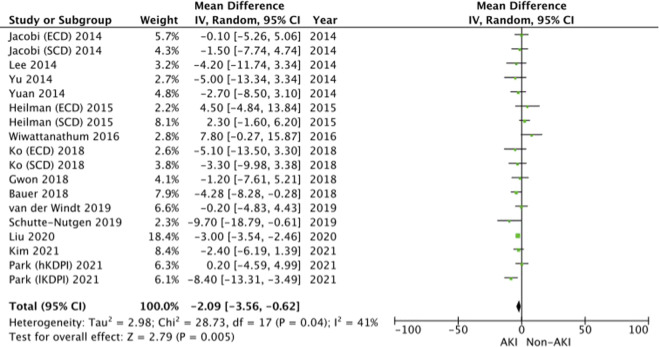
eGFR—Forrest plot.

14 studies [14, 19–22, 28–30, 35, 37, 39, 43–45] reported the eGFR at 12 or more months post-renal transplantation. The pooled results show similar eGFR levels between the AKI and non-AKI populations (WMD = −2.09, 95% CI = −3.56 to 0.62, I^2^ = 41%, Z = 2.79, *p* = 0.05) (Figure 6).

#### Length of Hospital Stay

**FIGURE 7:**
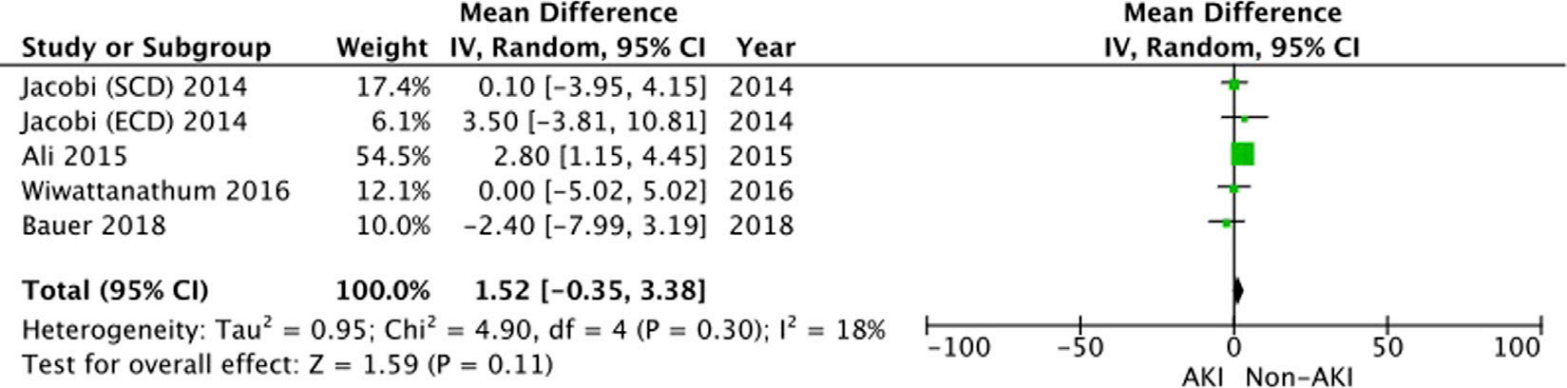
Length of hospital stay—Forrest plot.

4 studies [19, 32, 35, 37] reported the duration of hospitalisation in the 2 groups. These results demonstrate similar hospital stay length between the 2 populations (WMD = 1.52, 95% CI = −0.35 to 3.38, I^2^ = 18%, Z = 1.59, *p* = 0.11) (Figure 7).

## Discussion

AKI is highly common in the ITU population with over 35% of patients in ITU will developing AKI at some stage during their admission [24, 47]. Overall, the evidence from the single-centre, multi-centre and national registry studies included in this systematic review and meta-analysis supports transplanting these kidneys, potentially providing a significant boost to the prospective donor pool and reducing waitlist mortality. The UK transplant registry analysis found that 17% of the potential kidney donors had AKI. During a 10-year period (2003–2013) over 1,600 recipients received a kidney from a donor with AKI and had a functioning graft at 1-year post-transplant [13].

Kayler et al. [18] is the first large US transplant registry analysis investigating AKI donor kidneys. Their cohort of over 80,000 kidney transplant recipients was stratified based on the terminal serum creatinine levels (tSCr). Of note, high risk kidneys (deemed as those with tSCr >2 mg/dL) only represented 22% of the total pool of donors. This study demonstrated higher DGF rates in the AKI donor population, particularly in the ECD donors (36% in the SCD with AKI, 41% in the ECD with AKI, compared to 21% and 32% in the same groups when AKI was not present). This was the first study to demonstrate that a raised level of tSCr in the donor had no impact on the long term-graft survival from SCD kidneys. Our meta-analysis also supports the same long-term outcomes.

In the ECD group however, worse outcomes were recorded suggesting that parenchymal chronic changes could have a significant effect. This is in keeping with the existing knowledge suggesting that recovery of renal function is inversely proportional to age. A population of donors >65 years with comorbidities may have less likelihood of recovery of function [48, 49].

Kayler et al*.* [18] also highlights an important observation that kidneys with good urine output and no chronic changes on biopsy had comparable outcomes to those in which the SCr stabilised in terms of PNF, DGF and 1-year graft survival. One of the important limitations in this study is the reliance on tSCr without taking into consideration initial or peak levels. As kidneys with high tSCr are generally considered “high-risk,” this study was prone to selection bias. In addition, data on the donor urine output, RRT need, and perfusion technique, which are independent discriminating variables, have not been accounted for.

Rodrigo et al. [24] is the first study to apply the RIFLE (Risk, Injury, and Failure; and Loss, and End-stage kidney disease) criteria [50] to analyse the kidney damage. This classification considers the renal function dynamic as opposed to focusing on tSCr values. In this study, AKI donor kidney recipients had a higher risk of DGF, higher immediate creatinine levels and lower urine output. Importantly, these seem to normalise from 6 months onwards. The long-term graft function and 5-year graft survival were similar (58.4% AKI vs. 61.5% non-AKI kidneys). This study demonstrates that age, hypertension, a higher APACHE II score, hypotensive episodes, and length of ICU stay, are directly proportional with the chance of developing AKI. This is an important finding as the life expectancy and comorbidity status of the general population is on the rise. In this study, 85% of the donors had either traumatic head injury or a cerebrovascular accident (CVA), and this might not be representative of general ITU patient population but does probably represent standard donor population. In addition, it is important to note the limited population in the ‘failure’ category. Yu et al. [29] had a significantly higher cohort of patients in this category, and demonstrated no statistical difference in relation to PNF, DGF, acute rejection and renal function and graft survival. They observed that the risk of DGF rises exponentially with the increase in the AKI severity.

In contrast with Rodrigo et al. [24], Kolonko et al. [25] reported inferior immediate graft function in the AKI group (30% vs. 10%) and a higher risk of graft loss (28% vs. 7%). However, there was no substantial difference in the longer-term renal function (eGFR >12 months). These findings were also consistent with our meta-analysis.

The link between AKI and DGF was demonstrated by Farney et al. [26], 30% of the recipients of an AKI kidney being at risk of developing DGF compared to 13% in the non-AKI donor population. Although the study suggests a lower 3-year graft survival when DGF is present, there is no difference between the 2 populations (68% vs. 90% non-AKI with and without DGF vs. 89% and 91% AKI with and without DGF). The SCr levels are similar at 1- and 2-year post-transplantation across the entire cohort, reiterating the hypothesis that renal function recovery begins in the donor and continues post-transplantation. This study highlights the importance of knowing the baseline renal function as variation from it cannot be established in its absence.

In Jung et al. [27] the terminal serum creatinine (tSCr) was determined as an independent risk factor of DGF and slow graft function (19% in the AKI group vs. 5% in the non-AKI group). In accordance with the previous reported findings, the long-term allograft function and rejection-free survival do not significantly differ in this study. Jacobi et al. [19] demonstrated similar findings, although the study reported the lowest allograft survival rate in ECD population at 78%. Their subgroup analysis revealed that most graft losses were secondary to perioperative complications rather than due to the AKI status, which is an important confounding feature.

Lee et al. [51] was the first study to utilise the AKIN classification [52] as opposed to the previously used RIFLE criteria or tSCr. By applying the AKIN criteria, the results remained consistent with the existing knowledge demonstrating a higher rate of DGF in the AKI group (42% vs. 12%) and a non-inferior medium-term graft survival. Similarly, Ali et al. [32] demonstrated an exponential increase in DGF, 60% of the AKIN stage 3 population developing DGF vs. 25% in the non-AKI donor group.

Benck et al. [33], Hall et al. [34] and Heilman et al. [20] also reported comparable findings in terms of DGF, allograft function and survival. An important advantage of the latter study is that it only excluded kidneys with cortical necrosis or moderate-severe chronic changes on biopsy. If these criteria would be extrapolated, this study estimates that a further 31% SCD and 22% ECD kidneys in the US could become transplantable.

With the emergence of the KDIGO classification, the question about whether previous AKI classification systems are inferior arose. Kim et al. [36] addressed this by comparing the KDIGO and AKIN criteria and demonstrated that although KDIGO criteria has a better predictive value for DGF, both provide similar predictive value with respect to allograft function and survival.

van Der Windt [44] investigated the link between AKI kidneys and histology, demonstrating a similar degree of fibrosis on biopsies obtained 1-year post-transplantation, reiterating that recovery continues in the recipient. The limitation of this study lies in their cohort of mainly AKIN stage 1 kidneys, rendering the study underpowered to draw a conclusion regarding higher degrees of AKI.

In the UK, Boffa et al. [13] published a large national transplant registry analysis comprising of 11,000 donors. This is the first study in the literature demonstrating contrasting results in the rates of graft failure at 1 year compared to the previous studies. They have reported a reduction in 1-year graft survival in the AKI group by 2% (89% vs. 91%), however the clinical significance of this remains limited particularly if balanced against the annual death rate by remaining on the waiting list. This is in contradiction with our meta-analysis which found no significant difference in 1-year graft survival. Their results also showed that approximately 28% of kidneys were not utilised, and AKI stage 3 kidneys being 20 times more likely to be discarded. In contrast to the previous studies linking age with the likelihood of developing CKD, the Cox-regression analysis did not identify age as an independent risk factor. Caution was advised regarding utilisation of AKI stage 3 donors given the higher rates of DGF (three times greater vs. non-AKI) and PNF (9% vs. 4%). They have suggested counselling patients in regards to the risks and benefits of AKI kidneys when considering the utilisation of AKI stage 3 kidneys. *Bauer et al.* [37] employed this strategy successfully, showing that in their cohort, none of the patients refused transplantation from such kidneys.

In contrast, Liu et al. [45], a large registry-based, propensity-matched cohort study of over 25,000 recipients, showed that AKI status had no correlation with death-censored graft failure (HR 1.01; 95% CI 0.95–1.08) or all-cause graft failure (HR 0.97; 95% CI 0.93–1.02), across all AKI stages.

These findings were replicated by Pei et al. [46], which demonstrated that donor AKI stage did not negatively correlate with post-transplant outcome (allograft failure, mortality, acute rejection), except for DGF (44% in the AKI donor group vs. 26% in the non-AKI group). However, interpretation of this remains limited in high stage AKI, given the lower numbers in the AKI stage 3 category. This study demonstrated acceptable overall outcomes when transplanting kidneys from donors with AKI in line with previously published data [53].

Our meta-analysis has several limitations. Firstly, there is a considerable heterogeneity in the included studies, particularly when reporting DGF, acute rejection and allograft survival. This is unavoidable due to the population and methodological diversity [54]. This was accounted for by using a random-effects model when performing this meta-analysis.

Secondly, all the studies included in this meta-analysis were retrospective cohort studies. A particular drawback of retrospective studies is selection bias. A larger proportion of ‘lower risk’ AKI kidneys could have potentially been selected as acceptable for transplant, particularly in the early studies as the AKI donor profile was emerging. However, a randomised control trial addressing this would be logistically and ethically challenging to perform.

The large number of pooled donors (over 110,000), provide a good population size and renders our meta-analysis findings both representative and generalisable. There appears to be no significant difference in the odds of allograft survival (OR 0.95, 95% CI = 0.81–1.12, *p* = 0.54) between the two groups. This data should be interpreted cautiously as the included studies reported a mixture of death-censored and non-death censored graft survival over variable lengths of time (ranging from 12 to 120 months). Hazard ratios (HR) could not be calculated due to under-reporting of specific values in the literature. In addition, subgroup analyses stratifying the risk according to the AKI stage or determining if there are different outcomes between current and recovering AKI was also not reported in most of the studies included in this meta-analysis.

The criteria utilised to define AKI was also inconsistent. This is unavoidable due to the temporal evolution of these classification systems (RIFLE, AKIN, KDIGO). However, multiple studies demonstrate no significant differences in the prognostic value of these systems [36, 55–58].

As the acceptable AKI kidney donor profile is developing, future research is required to determine the long-term outcomes, risk stratification and optimal selection methods of these kidneys. Development of accurate AKI biomarkers to predict post-transplant outcomes would aid the selection of AKI donor kidneys [59–61]. Novel perfusion strategies are also increasingly being utilised in the assessment and pre-conditioning of organs. Normothermic regional perfusion (NRP) is a promising emerging technique which could provide functional assessment and ischaemic pre-conditioning of donor organs. Early existing data supports this, demonstrating that NRP reduces the rates of DGF and PNF in the post-DCD transplantation population [62–64].

## Conclusion

This study demonstrates that transplanting kidneys from donors with AKI can lead to satisfactory outcomes. The rates of DGF are higher in this population but does not seem to impact long-term allograft function and survival. With higher AKI stage kidneys, a degree of caution is advised, however, these organs could be judiciously utilised discussing the potential benefits and risks on an individual basis. Donor kidneys with AKI remain an underutilised pool of resource which could help bridge the existing gap between supply and demand, ultimately improving outcomes and survival for transplant waitlisted patients.

## Figures and Tables

**Table:** 

**ACR**	Albumin:Creatinine Ratio
**ADQI**	Acute Dialysis Quality Initiative
**AKI**	Acute Kidney Injury
**AKIN**	Acute Kidney Injury Network
**AR**	Acute Rejection
**ARF**	Acute Renal Failure
**AS**	Allograft Survival
**CEBM**	(Oxford) Centre of Evidence-Based Medicine
**CIT**	Cold Ischaemia Time
**CKD**	Chronic Kidney Disease
**DBD**	Donation after Brainstem Death
**DCD**	Donation after Circulatory Death
**DGF**	Delayed Graft Function
**ECD**	Expanded Criteria deceased Donors
**ESKD**	End-Stage Kidney Disease
**(e)GFR**	(estimated) Glomerular Filtration Rate
**KDIGO**	Kidney Disease: Improving Global Outcomes
**KDPI**	Kidney Donor Profile Index
**NHS**	National Health Service
**NHSBT**	NHS Blood and Transplant
**NICE**	National Institute for Health and Care Excellence
**NOS**	Newcastle-Ottawa Scale
**NRP**	Normothermic Regional Perfusion
**PNF**	Primary Non-Function
**PRISMA**	Preferred Reporting Items for Systematic Reviews and Meta-Analyses
**pSCr**	peak Serum Creatinine
**QoL**	Quality of Life
**RIFLE**	Risk-Injury-Failure-Loss-End stage kidney disease
**RRT**	Renal Replacement Therapy
**SaBTO**	Advisory Committee on the Safety of Blood, Tissues and Organs
**SCD**	Standard Criteria deceased Donors
**SCr**	Serum Creatinine
**tSCr**	terminal Serum Creatinine
**UO**	Urine Output
